# Death from Transfusion-Transmitted Anaplasmosis, New York, USA, 2017

**DOI:** 10.3201/eid2408.172048

**Published:** 2018-08

**Authors:** Ruchika Goel, Lars F. Westblade, Debra A. Kessler, Maroun Sfeir, Sally Slavinski, Bryon Backenson, Linda Gebhardt, Kathleen Kane, Jeffrey Laurence, Douglas Scherr, James Bussel, J. Stephen Dumler, Melissa M. Cushing, Ljljana V. Vasovic

**Affiliations:** Weill Cornell Medicine, New York, New York, USA (R. Goel, L.F. Westblade, M. Sfeir, J. Laurence, D. Scherr, J. Bussel, M.M. Cushing, L.V. Vasovic);; New York-Presbyterian Hospital, New York (R. Goel, K. Kane, M.M. Cushing);; New York Blood Center, New York (D.A. Kessler);; New York City Department of Health and Mental Hygiene, Wadsworth, New York, USA (S. Slavinski, B. Backenson);; Wadsworth Center, New York State Department of Health, Albany, New York, USA (L. Gebhardt);; Uniformed Services University of the Health Sciences, Walter Reed National Military Medical Center, Bethesda, Maryland, USA (J.S. Dumler)

**Keywords:** red blood cell transfusion, anaplasmosis, transfusion-transmitted infectious disease, Anaplasma phagocytophilum, leukoreduction, bacteria, New York, United States

## Abstract

We report a death from transfusion-transmitted anaplasmosis in a 78-year-old man. The patient died of septic shock 2 weeks after a perioperative transfusion with erythrocytes harboring *Anaplasma phagocytophilum*. The patient’s blood specimens were positive for *A. phagocytophilum* DNA beginning 7 days after transfusion; serologic testing remained negative until death.

Human granulocytic anaplasmosis (HGA) is caused by the obligate intracellular gram-negative bacterium *Anaplasma phagocytophilum* and transmitted primarily by ticks of the genus *Ixodes* (*1*–*3*). Although HGA is largely asymptomatic, manifestations can range from fever, myalgia, headache, or malaise to life-threatening complications in elderly or immunocompromised patients (*2*,*4*). The prevalence of anaplasmosis is increasing in the United States (*4*). Blood components are not currently screened for *A. phagocytophilum*. Because *A. phagocytophilum* is found predominantly within granulocytes, leukoreduction lowers the risk of transfusion-transmitted anaplasmosis (TTA) by reducing the level of the organism by 300-fold (*5*). To date, 9 cases of TTA attributed to erythrocytes and platelets, including leukoreduced units, have been reported (*5*–*7*).

We report a fatality associated with TTA in an elderly man in New York, New York, USA. The Weill Cornell Medical Center institutional review board (New York, NY, USA) reviewed this case report.

## Case Report

A 78-year-old man with homozygous factor XI deficiency and an extensive medical history, including coronary artery disease, congestive heart failure, diabetes, and chronic kidney disease, was admitted in April 2017 for transurethral resection of the bladder as the result of a urologic malignancy. He had a history of anemia (preoperative hemoglobin 9.9 g/dL; reference range 14–17.5 g/dL); darbepoietin had been prescribed to him as an outpatient, with limited compliance. As an inpatient, he received a diagnosis of iron deficiency; his serum iron level was 13 (reference range 65–175 μg/dL). Minimal blood loss occurred during surgery, but the patient’s early postoperative course was complicated by recurrent hematuria and symptomatic anemia. Twelve units of leukoreduced, irradiated erythrocytes were required. The patient also received 14 plasma transfusions to keep his factor XI levels above the hemostatic threshold.

**Figure 1:**
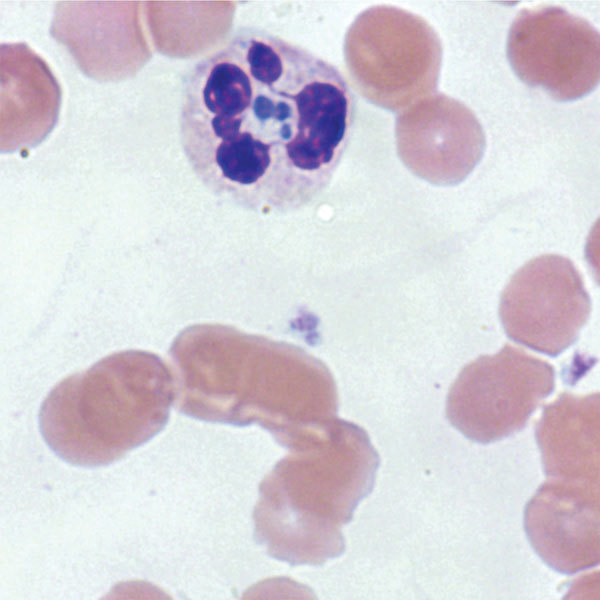
*Anaplasma phagocytophilum* morulae observed on peripheral blood smear from patient in whom anaplasmosis infection developed after a blood transfusion, New York, New York, USA. Intracytoplasmic inclusions (morulae) were first seen 15 days after the patient was transfused with an infected erythrocyte unit, leading to a diagnosis of human granulocytic anaplasmosis later confirmed by PCR (original magnification ×1,000 [oil immersion]).

**Figure 2:**
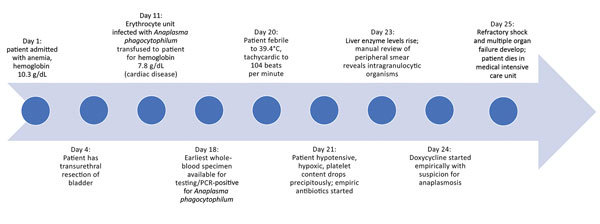
Timeline of patient’s hospitalization for anemia followed by *Anaplasma phagocytophilum* infection, New York, New York, USA.

On day 20 of admission, the patient unexpectedly became febrile and developed tachycardia and hypotension. The patient had previously been afebrile and was not taking empiric antimicrobial drugs. Cultures were drawn, and the patient was given broad-spectrum antibacterial drugs (piperacillin/tazobactam, 4.5 g intravenously [IV] every 12 h; and vancomycin, 1.5 g IV every 24 h). Despite antibacterial drug coverage and negative blood, urine, and throat cultures, the patient continued to be febrile and decompensated with dyspnea, hypoxia, and an elevation in troponin. He subsequently developed thrombocytopenia, leukopenia, and transaminitis. On day 4 of fevers, clusters of bacteria within neutrophils, consistent with *A. phagocytophilum*, were seen on peripheral smear (the inclusions were not identified on prior smears, even upon retrospective review) (Figure 1). Doxycycline (100 mg IV every 12 hours) was started immediately, pending *Anaplasma*/*Ehrlichia* serology. The patient went into refractory shock, developed multiple organ failure, and died 24 hours after initiation of doxycycline (Figure 2).

Although the season was early spring, the likelihood of tick-bite transmission was extremely low, as the patient had been homebound for weeks before admission and had no pets (thus eliminating tick exposure via household pets). We pursued an investigation for transfusion-transmitted infection. Nucleic acid–based testing on available patient samples and segments from all transfused erythrocyte units was performed by the New York State Department of Health (NYS DOH). One of 12 donor unit segments tested positive by PCR for *A. phagocytophilum* DNA. This erythrocyte unit was whole blood derived with 1 co-component from the donation: a plasma unit that had already been transfused with no adverse reactions. The NYS DOH identified *A. phagocytophilum* in each of the patient’s blood specimens beginning 7 days after the implicated transfusion (the first patient specimen available for testing) (*8*). However, immunofluorescence assay results for *A. phagocytophilum* antibodies were negative until death (*9*). 

At autopsy, fresh tissue from the spleen and bone marrow tested positive for *A. phagocytophilum* by PCR. Results of immunohistochemistry testing of fixed tissue obtained from lung, heart, liver, spleen, and kidney were positive for *A. phagocytophilum* (*9*). This transfusion-related fatality was reported to NYS DOH and the US Food and Drug Administration.

The implicated erythrocyte unit had been stored for 22 days before transfusion; routine testing for *Babesia microti* performed under an investigational new drug application protocol was negative at the time of donation. During investigation of the transfusion-transmitted infection, the donor reported a rash and headache but had no fever around the time of donation. The donor’s doctor had empirically treated him for Lyme disease, but exact details of the treatment were not available. The donor did not report a postdonation illness despite a standard reminder during donation to report such information. The donor was deferred from blood donation for 90 days.

This patient’s diagnosis was HGA, a tickborne illness previously called human granulocytic ehrlichiosis (*10*). HGA should be included in the differential diagnosis of fever after tick bites in the upper Midwest and northeastern United States and in northern California (*1*,*3*,*4*,*11*). HGA symptoms typically appear 5–21 days after a tick bite and manifest as fever (75%–100%), myalgia (75%), headache (83%), and malaise (97%). Laboratory test abnormalities such as thrombocytopenia (79%), leukopenia (60%), and transaminitis (91%) increase diagnostic specificity (*3*). Although HGA can be asymptomatic in healthy adults, it can have life-threatening consequences in the elderly, patients with immune-compromising conditions (e.g., diabetes or cancer), or patients taking immunosuppressive medications (*2*). Symptoms of severe HGA may include renal failure, respiratory distress syndrome, toxic shock–like syndrome, pneumonia, and disseminated intravascular coagulation or sepsis-like syndrome. Overall, 31% of patients are hospitalized, and 7% require intensive care. The case-fatality rate is 0.6%, with a 16-fold increase in relative risk for death if infection occurs in a patient with an immunosuppressive condition. Anaplasmosis and other tickborne infections should be considered in a differential diagnosis of sepsis, especially in posttransfusion cases, when patients are refractory to routine broad-spectrum antimicrobial drugs. Standard culture techniques will not detect causative organisms and the infections require directed therapy (e.g., doxycycline) not usually included in standard empiric treatment.

*A. phagocytophilum* resides intracellularly (primarily in neutrophils), can cause subclinical infection, and can survive refrigeration (*5*,*12*); thus, it meets the criteria for an erythrocyte transfusion-transmitted infection. As of May 2018, a total of 9 cases of TTA have been reported: 7 attributed to erythrocyte units (5 leukoreduced), 1 involving a leukoreduced apheresis platelet unit, and 1 from whole blood–derived platelets. Leukoreduction leads to a 300-fold reduction in *A. phagocytophilum* bacteremia but does not eliminate the risk of infection (*13*). The longest survival of *A. phagocytophilum* previously reported in refrigerated conditions was 18 days (*5*,*7*,*12*); in our case, we report a prolonged survival of *A. phagocytophilum* in a refrigerated erythrocyte unit (22 days).

## Conclusions

This case illustrates a challenge in transfusion medicine as a result of limitations of infectious disease screening of the blood supply. No Food and Drug Administration–licensed tests exist for screening donated blood for *A. phagocytophilum.* Serologic or nucleic acid testing of donors is considered unnecessary because transfusion transmission of *A. phagocytophilum* is an extremely rare event. A history of tick bites is generally not obtained from donors because it is neither sensitive nor specific (*14*). 

Pathogen reduction would likely have prevented HGA transmission in this case. However, only pathogen-reduced plasma and platelets are currently available. Pathogen-reduced erythrocytes remain under development. The most prudent approach to reduce the risk of transfusion-transmitted anaplasmosis is by the judicious use of blood components and the strict avoidance of unnecessary transfusions in adherence with published guidelines (*15*). This fatality from transfusion-transmitted anaplasmosis is a reminder of the serious residual risks from transfusions, as well as the increasing prevalence of *A. phagocytophilum*.
