# Correction: Vol. 24, No. 8

**DOI:** 10.3201/eid2409.C12409

**Published:** 2018-09

**Keywords:** errata, erratum, correction

The author list was incorrect in Death from Transfusion-Transmitted Anaplasmosis, New York, USA, 2017 (R. Goel et al.), and a name was missing from the acknowledgments. Melissa M. Cushing should have been listed as senior author. Ljljana V. Vasovic provided assistance with the article. The article has been corrected online (https://wwwnc.cdc.gov/eid/article/24/8/17-2048_article).

